# Supramolecular Hybrid Material Based on Engineering Porphyrin Hosts for an Efficient Elimination of Lead(II) from Aquatic Medium

**DOI:** 10.3390/molecules24040669

**Published:** 2019-02-14

**Authors:** Chahrazad El Abiad, Smaail Radi, Maria A. F. Faustino, M. Graça P. M. S. Neves, Nuno M. M. Moura

**Affiliations:** 1 Laboratory of Applied Chemistry and Environment (LCAE), Department of Chemistry, Faculty of Sciences, University Mohamed Premier, Oujda 60000, Morocco; elabiad.1987@gmail.com; 2 QOPNA & LAQV-REQUIMTE, Department of Chemistry, University of Aveiro, 3810-193 Aveiro, Portugal; faustino@ua.pt (M.A.F.F.); gneves@ua.pt (M.G.P.M.S.N.)

**Keywords:** porphyrinoids, hybrid material, adsorption, heavy metal cation, selectivity, lead

## Abstract

Porphyrins show great promise for future purification demands. This is largely due to their unique features as host binding molecules that can be modified at the synthetic level, and largely improved by their incorporation into inorganic based materials. In this study, we assessed the efficacy of a hybrid material obtained from the immobilization of 5,10,15,20-tetrakis(pentafluorophenyl)-porphyrin on silica surface to remove Pb(II), Cu(II), Cd(II), and Zn(II) ions from water. The new organic-inorganic hybrid adsorbent was fully characterized by adequate techniques and the results show that the hybrid exhibits good chemical and thermal stability. From batch assays, it was evaluated how the efficacy of the hybrid was affected by the pH, contact time, initial metal concentration, and temperature. The adsorption kinetic and isotherms showed to fit the recent developed fractal-like pseudo-second-order model and Langmuir–Freundlich model respectively. The highest adsorption capacities for Pb(II), Cu(II), Cd(II), and Zn(II) ions were 187.36, 125.17, 82.45, and 56.23 mg g^−1^, respectively, at pH 6.0 and 25 °C. This study also shows that metal cations from real river water samples can be efficient removed in the presence of the new adsorbent material.

## 1. Introduction

The high number of applications involving potentially toxic metal ions is being associated to an increase of metallic substances present in the environment. The beneficial impact of the industrial revolution has been in many aspects accompanied by a negative impact in the environment. For instance, the direct discharge of industrial effluents in watercourses is making them unsuitable for living organism consumption and even for domestic and irrigation use. Heavy metal ions are pointed out as one of the most dangerous inorganic contaminants present in polluted water and are being associated to many health effects [1,2,3]. Long-term exposure to potentially toxic metals ions, such as Pb(II), Cu(II), Cd(II), or Zn(II) are being associated with liver and kidney damage, lung cancer, reduction in hemoglobin formation, emphysema, hypertension, testicular atrophy diseases or itching [4,5].

Heavy metal ions are highly poisonous and can be found in discharged waters from several industries, such as coating, storage batteries, and aeronautical. Furthermore, it may leach into drinking water from certain types of plumbing materials. These metal ions can be responsible for serious health effects like hypertension, muscle and joint pain, irritability, memory or concentration problems, kidney failure, and nervous diseases [3,6]. Therefore, in recent years, a high number of studies have been related with the removal of potentially toxic metals from contaminated water, namely industrial wastewater. In addition, an important investment has been dedicated to improve techniques related with wastewater treatment namely conventional physicochemical methods, such as reverse osmosis [7], flotation [8], coagulation [9], ion exchange [10], chemical precipitation [11], membrane filtration [12], and adsorption [13,14]. Between them, adsorption is considered a promising and widely used approach. Among the different materials capable of capturing metal ions, aqueous solutions are included activated carbon [15], fly ash [16], microbial biomass [17], and biomass materials [18,19]. However, these materials have shown some disadvantages, such as weak chemical bonds, limited removal efficiency, high cost, and low mechanical and thermal stability [20].

On the other hand, materials based in silica gel have many advantages, due to their good mechanical and thermal stability, high porosity, and good swelling resistance in the presence of different solvents. Additionally, their surfaces can be easily modified by using different approaches [21,22,23].

Recently, an important topic of research has been related to the development of silica gel based organic-inorganic hybrid materials for the removal of heavy metal ions [24,25,26,27,28,29]. Silica gel is considered an ideal inorganic solid matrix, not only due to its surface property, but also to its relative simplicity, when compared with polymer resins. So, a high number of chemically modified silica gels have been prepared, and in general, their adsorption properties are mainly dependent on the presence of active donor atoms, like sulfur, oxygen and nitrogen, of the incorporated organic units [30,31]. 

In this context, porphyrins, well-known by their highly intense colors and key roles in basic mechanisms of living organisms are considered a special class of heterocyclic ligands, due to their unique properties, namely a high ability to coordinate a wide range of metal ions affording stable complexes [32,33,34,35,36,37,38]. In fact, the porphyrin capacity to recognize specific analytes has attracted the attention of the scientific community in the chemical sensing field, due to a possible selectivity enhancement involving supramolecular chemistry concepts [34,39]. These porphyrins can host, through their backbone and inner core, analytes according to various mechanisms, including hydrogen bonds, Van der Waals forces, π-π interactions, and coordination [33,40]. In particular, the inner nitrogen donor atoms are able to interact with a wide range of metal ions, and therefore, allow for their detection or elimination with high efficiency [41].

The access to porphyrins with adequate properties for a special application can be easily tuned by structural modifications at different stages of the porphyrin synthesis [42]. One of the strategies can involve the post-functionalization of the porphyrin macrocycle by attachment to additional groups. In this context, 5,10,15,20-tetrakis(pentafluorophenyl) porphyrin (**H_2_TF_5_PP**), has revealed to be a versatile template to afford new porphyrin derivatives for a wide range of applications [43,44,45,46,47,48]. In fact, the reaction of **H_2_TF_5_PP**, or of the corresponding metal complexes, with nucleophiles is considered a simple and general strategy to afford *meso*-tetraarylporphyrins functionalized at the *para* position of their *meso*-aryl groups with electron-donating substituents. In addition, the selective substitution of the *para*-F atoms occurs frequently with high yield [49].

In this paper, the synthesis and the characterization of an inorganic-organic hybrid material obtained by the covalent attachment of **H_2_TF_5_PP** on silica, and its use in potentially toxic metal ions removal, is assessed by considering the importance of developing efficient metal adsorbents. All parameters that may affect the adsorption efficiency of metal ions (e.g., pH, contact time, and concentration) have been studied. The ability of the adsorbent to remove metal cations from real water samples, as well as its reusability, was evaluated and compared with the extracted quantities of metals ions determined by atomic absorption measurements.

## 2. Results and Discussion

### 2.1. Synthesis and Characterization of the Organic-Inorganic Hybrid Material SiTF_5_PP

**Scheme 1:**
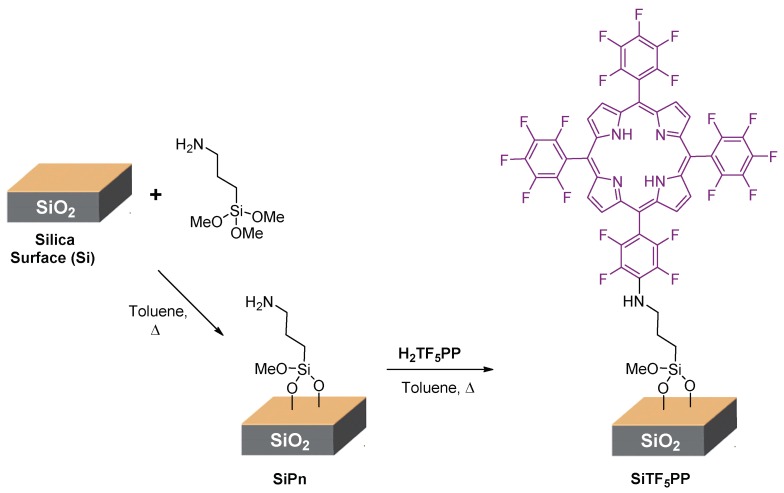
Synthetic methodology used to prepare the inorganic-organic hybrid **SiTF_5_PP**.

The synthetic procedure giving rise to the organic-inorganic hybrid material **SiTF_5_PP** is outlined in Scheme 1. The first step involved the decoration of the activated silica Si with amino groups, through its reaction with 3-aminopropyltrimethoxysilane in toluene. The second step, comprising the reaction between the obtained amino functionalized silica material **SiPn** and the porphyrinic ligand 5,10,15,20-tetrakis(pentafluorophenyl)porphyrin (**H_2_TF_5_PP**), was performed in refluxing toluene for 48 h.

The properties and morphology of the synthesized adsorbent material were studied by using the appropriate techniques like elemental analysis (EA), attenuated total reflection–fourier transform infra-red (ATR-FTIR), powder X-ray diffraction (XRD), scanning electron microscopy (SEM) and transmission electron microscopy (TEM), ^13^C solid-state CPMAS NMR, Brunauer-Emmett-Teller (BET) surface area and Barrett-Joyner-Halenda (BJH) analysis, thermogravimetric analysis (TGA), and solid-state UV-Vis.

The success of the two synthetic steps was clearly confirmed by elemental analysis (see Table S1 in Electronic Supplementary Information, ESI). The presence of carbon and nitrogen in **SiPn** indicates that the aminopropylation reaction was successfully accomplished, since neither element is present in native silica. The increase in the content of carbon in **SiTF5PP,** when compared with **SiPn** (C, 8.00 %; N, 1.53 % verus C, 5.03 %; N, 1.63 %), indicates that the porphyrin was attached to **SiPn**. The ATR-FTIR spectra of the starting material Si, the materials **SiPn** and **SiTF_5_PP** are shown in Figure S1 in the ESI. For Si, the broad and intense peak at *ca* 3462 cm^−1^ can be ascribed to the O–H stretching vibration of the silanol group. The intense bands at 1638 cm^−1^ and at 1096 cm^−1^ are attributed to the stretching vibration of Si–OH and Si–O–Si, respectively, while the bands at *ca.* 972 cm^−1^ result from the Si–O vibration [50].

In the spectrum of **SiPn**, new bands at 2979 cm^−1^ and 1563 cm^−1^ are observed, which can be attributed to *_ѵ_*(C-H), and *_ѵ_*(NH_2_), stretching vibrations, respectively [51]. These bands are indicative of the presence of 3-aminopropylsilane units on silica particle surface. The ATR-FTIR spectra of the hybrid material **SiTF5PP** reveals the disappearance of the band at 1563 cm^−1^, due to the reaction of the primary amine groups (-NH_2_), and the appearance of new bands in the range 1503–1450 cm^−1^ and at 802 cm^−1^. This is due to the valence vibrations of C–C bonds of pentafluorophenyl substituents and of the porphyrinic macrocycle [52]. Moreover, new characteristic bands around 1532 cm^−1^ and 1468 cm^−1^ resulted from *_ѵ_*(C=N), and *_ѵ_*(C=C) vibrations, respectively. 

**Figure 1:**
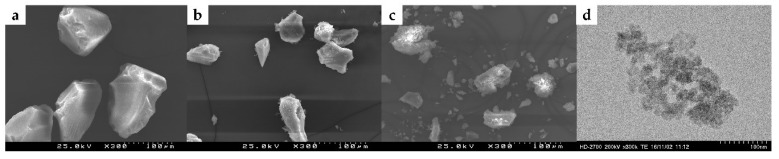
SEM images of free silica **Si** (**a**), **SiPn** (**b**) and **SiTF_5_PP** (**c**) and scanning transmission electron microscopy (STEM) image of **SiTF_5_PP** (**d**).

The modification in morphological features, after the silica surface functionalization, was clarified using SEM (Figure 1). The surface of the native silica **Si**, that was initially smooth, becomes rough after functionalization. Moreover, the immobilized surface **SiTF_5_PP** tends to be partially aggregated due to the presence of a high amount of the organic component. This morphology was confirmed using scanning transmission electron microscopy (STEM), which is very sensitive to variations of surface structure. Indeed, a rough surface is clearly highlighted in the STEM micrograph as shown in Figure 1d. These results confirm the successful anchorage of the porphyrin unit at silica surface, and it is known that a rough surface can be beneficial to metal ion adsorption.

The presence of the porphyrin moiety in the final hybrid material **SiTF_5_PP** was also confirmed by ^13^C solid-state CPMAS NMR (Figure S2 in ESI). The ^13^C spectrum of **SiPn** shows signals at *δ* 9.75, *δ* 22.06, and *δ* 42.66 ppm, due to the resonances of the aliphatic propyl carbons Si–CH_2_, –CH_2_–, and N–CH_2_, respectively. In the same region “a”, the signal at *δ* 50.96 ppm is assigned to the resonance of the non-substituted methoxy group (-OCH_3_). The additional peaks that appear in the ^13^C spectrum of **SiTF_5_PP** in the low field region are categorized as “b” between *δ* ≅ 106 and *δ* ≅ 165 ppm, due to the aromatic carbon resonances of the porphyrin macrocycle. 

**Figure 2:**
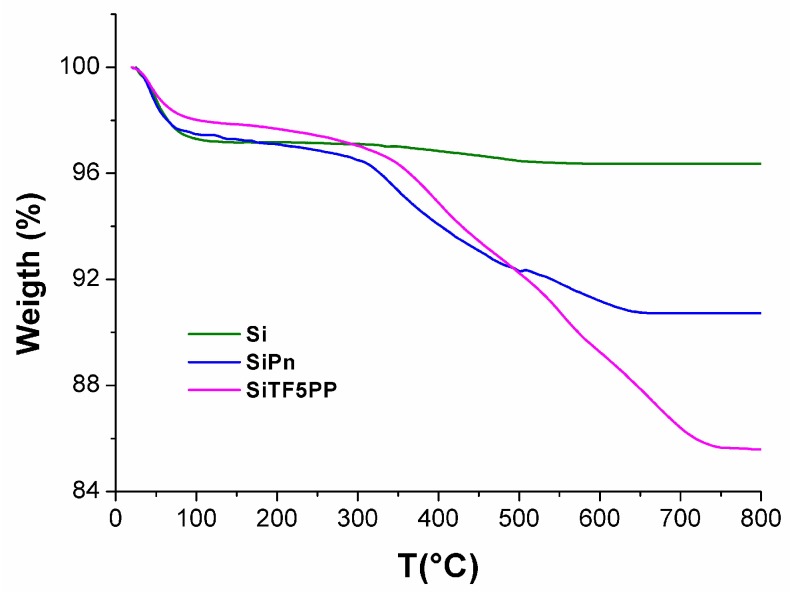
Thermogravimetric curves of **Si**, **SiPn** and **SiTF_5_PP**.

The thermal stability of the new hybrid material **SiTF_5_PP** was evaluated by thermogravimetric analysis, and it was compared with the thermal stability of the precursors **Si** and **SiPn** (Figure 2). The TGA of all the studied materials exhibited a mass loss below 110 °C, assigned to the loss of the physically adsorbed of water [53]. The native **Si** shows a second mass loss up to 800 °C assigned to condensation of free silanol groups bonded to the surface [54]. Similar to the native silica (**Si**), **SiPn** and **SiTF_5_PP** also show additional mass losses, due to the decomposition of the organic groups decorating the silica surface [55,56,57]. These data indicated that the adsorbent **SiTF_5_PP** has good thermal stability and the immobilization of the porphyrin macrocycle was accomplished with success.

**Figure 3:**
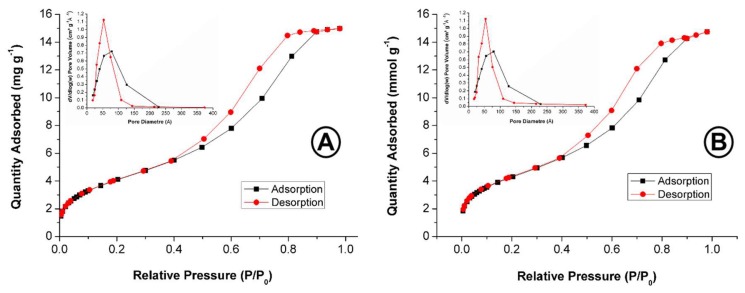
Nitrogen adsorption-desorption isotherm at 77 K plots of **SiPn** (**A**) and **SiTF_5_PP** (**B**). Inset is given the respective BJH pore size distribution curves.

In order to evaluate how the surface and porosity of the activated silica were changed after the introduction of 3-amino-propyl and **H_2_TF_5_PP**, the surface area SBET (Brunauer-Emmett-Teller), and the pore diameters and pore volumes of native silica, **Si**, **SiPn,** and **SiTF_5_PP,** were determined using nitrogen adsorption−desorption isotherms, and Barrett-Joyner-Halenda (BJH) methods, respectively (Figure 3) [58,59]. It is important to highlight that the isotherm curves for all materials (Figure 3) are type IV according to the classification of IUPAC, showing that partial pressures (P/P_0_ > 0.4) are a pronounced hysteresis, which is clear evidence of the mesoporous nature of the materials. In addition, the hysteresis loops are type H2, thereby indicating a uniform pore diameter distribution. The median pore size distribution (<50 Å) also confirms the uniform framework mesoporosity of the materials. Table 1 summarizes the physical parameters calculated from nitrogen adsorption-desorption isotherms. It was observed that **SiPn** decreased in surface area, total pore volume, and pore diameter compared with the initial **Si**. These results can be justified by taking into account the introduction of the organic functionalities into the mesoporous channels, which may diminish the pore size and increase the density of the material [54,60,61,62]. The increase in the surface area of **SiTF_5_PP**, relative to **SiPn,** is presumably due to the increasing surface roughness, as it was verified by SEM imaging. In addition, the pore volumes and the pore diameters remain unchanged, suggesting that the grafted porphyrins are located, not inside the pores, but rather on the outer surface, which confirms its further increase.

The XRD pattern of the **SiTF_5_PP** hybrid has been studied in the 2θ range of 5 to 50 and was compared with the XRD patterns of the activated silica **Si,** and of the aminopropyl functionalized silica **SiPn**. The XRD patterns summarized in Figure S3 (ESI) indicate that the materials prepared had maintained the mesoporous structure without significant impairment after modification. All diffractograms show the characteristic broad peak at low 2θ angle range due to the pore family (100) indicating a well-ordered type material [63]. Additionally, there is another weak broad peak (110) at a high 2θ angle (at about 23°), which is typical of amorphous silica, without peaks being assignable to any crystalline phase [63]. No significant changes on silica functionalization were observed, but as already indicated for other hybrid materials [64], the XRD peak intensity decreased in the case of the grafted samples, when compared to the initial silica. These results prove that the successful functionalization of the silica surface occurs mainly inside the mesopore channels.

**Figure 4:**
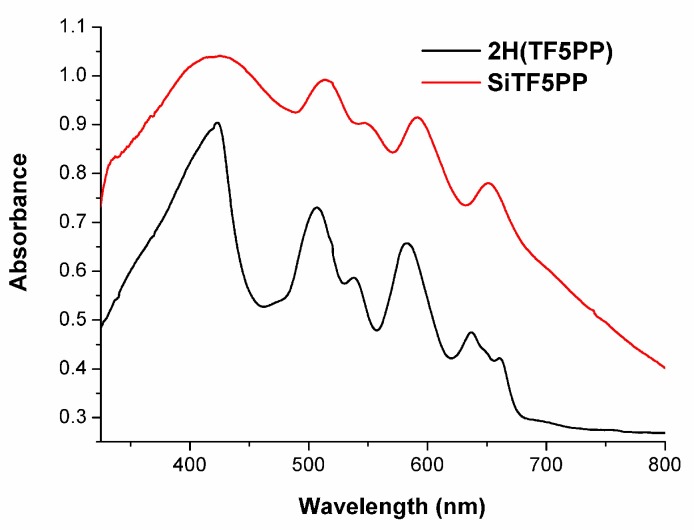
Solid-state UV-Vis spectra of **H_2_TF_5_PP** and of the hybrid **SiTF_5_PP**.

The successful anchorage of **H_2_TF5PP** to the matrix functionalized with amino groups was also confirmed by solid-state UV-Vis spectrophotometry. The UV-V is the absorption spectrum of the hybrid material **SiTF_5_PP,** which shows the typical broad absorption bands of the free-base porphyrin **H_2_TF_5_PP** in the solid state (Figure 4). The band broadening and the baseline elevation, observed in the solid UV-Vis of the hybrid, **SiTF_5_PP,** are due to the presence of solid-state interactions between the macrocycle and the silica [65].

The chemical stability of the newly synthesized hybrid **SiTF_5_PP** was investigated in the pH range 1 to 7, in acidic and buffer solutions. After acid treatment, the material structure remains unchanged as it was examined by elemental analysis (% C = 8.00 ± 0.20). The high stability presented by the attached organofunctional substituent is presumably due to the pendant chain responsible by the linkage between the amine and the **H_2_TF_5_PP** into the silica surface. It has been reported that when the length of the hydrocarbon bridge contains more than two methylene groups, the cleavage of Si–C bond is minimized under acidic medium, since these longer chains do not hold a functional handle that can undergo β-elimination of the Si cation [66,67].

### 2.2. Metal Cations Adsorption

#### 2.2.1. Effect of pH

**Figure 5:**
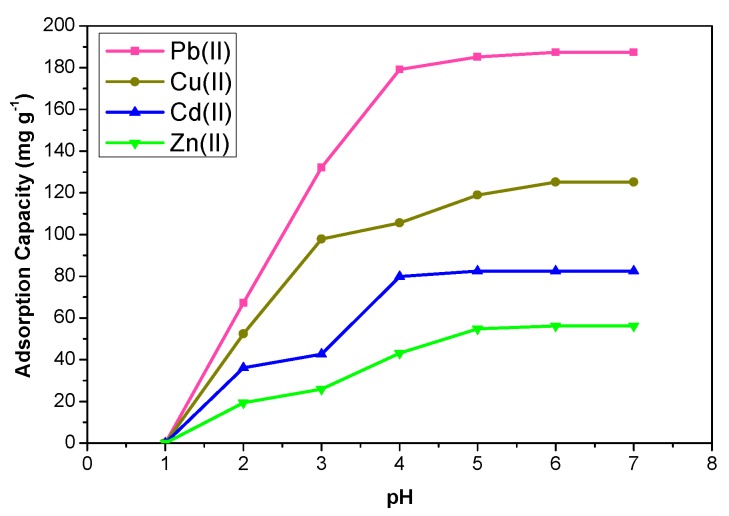
Effect of pH on the adsorption of studied metal ions on **SiTF_5_PP**. Adsorption dose: V = 10 mL, m = 10 mg of **SiTF_5_PP** at optimum concentration (187.36 ppm in each case), t = 30 min and 25 °C. Lines just combine the points.

It is well known that the pH value is an important factor affecting the removal of cations from aqueous solutions. The pH dependence of metal sorption is related to the chemistry of the metal in solution and also with the ionization state of the functional groups present in adsorbent, which can affect the availability of binding sites. Under this context, the adsorption capability of **SiTF5PP** towards Pb(II), Cu(II), Cd(II) and Zn(II) was determined in aqueous solutions with pH ranging between 1 and 7 as it is summarized in Figure 5. 

The results showed that at lower pH values, the metal cation retention is not significant, since the porphyrinic receptor is in its dicationic form and consequently unable to coordinate the metal cation. However, the data show that the deprotonation favors the adsorption and the best retention values were observed at pH 6–7 for the metal cations Pb(II), Cu(II), Zn(II), while for Cd(II) occur in the range 5–7. At pH values higher than 7, it is difficult to distinguish between the M(II) hydrolyzed or adsorbed, due to the precipitation of hydroxides, resulting from the metal ion hydrolysis. So, the other studies concerning the parameters that can affect the sorption efficiency of the metal ions were performed at pH 6.

#### 2.2.2. Effect of Contact Time and Adsorption Kinetics

**Figure 6:**
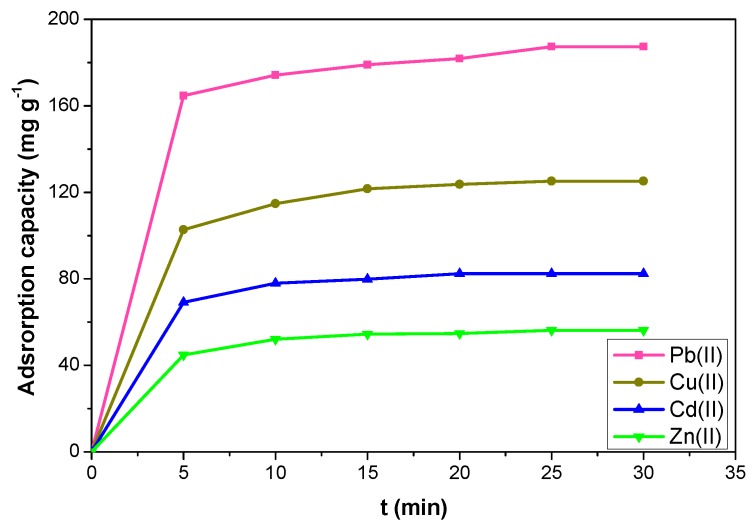
Effect of contact time on **SiTF_5_PP** adsorption capacity towards Pb(II), Cu(II), Cd(II), and Zn(II). Adsorption dose: V = 10 mL, m = 10 mg of **SiTF_5_PP**, at optimum pH (pH = 6), 25 °C and optimum concentration (187.36 ppm in each case). Lines just combine the point.

The results depicted in Figure 6, show that the adsorption efficacy of **SiTF_5_PP** towards the different metal ions is strongly dependent on its contact time with the analytes until the equilibrium conditions are attainment. The effect of contact time on the adsorption of Pb(II), Cu(II), Cd(II), and Zn(II) by **SiTF_5_PP** was studied by batch experiments, and the kinetic curves show a fast adsorption in the first 5 min reaching a plateau after about 25 min of contact. These prompt interactions with the free metal cations present in aqueous solution suggest that the porphyrinic units are adequately oriented and accessible, allowing the required incorporation of the metal cation in the macrocycle inner core. 

The superior efficacy of **SiTF_5_PP,** when compared with a previous material obtained from the immobilization of 2-formyl-5,10,15,20-tetraphenylporphyrin in silica (**SiNTPP**), previously described by us [68], (see Table 7) can be a consequence of the linkage position (β-pyrrolic *versus meso*-position) combined with the electronic features of the *meso*-substituents (pentafluorophenyl *versus* phenyl groups). 

Usually, the coordination of porphyrins with divalent metals occurs via the initial formation of a sitting-atop (SAT) porphyrin complex [69], where the metal ion interacts with the macrocycle inner nitrogen atoms without the simultaneous deprotonation of the *N*−H group. Depending on the size of the metal ion and on its spin multiplicity, a further step can happen involving the deprotonation of the two *N*−H groups, which is accompanied by the metal incorporation into the macrocycle core. The different metal selectivities can be justified, by considering the activation energy of the rate-limiting step for each metal ion [69,70].

So, in the case of **SiTF_5_PP**, the mesomeric electron-donor (+M) effect of the fluorine atoms can play a crucial role in the initial step by increasing the electronic density present on the non-protonated nitrogen atoms (see Figure S4, ESI). On the other hand, the fluorine inductive electron-withdrawing effect cans beneficiate the loss of two *N*-H protons of the second step. 

So, the great benefit of porphyrinic derivatives is the easy adaptation of their core to the metal ion size. These macrocycles are able to respond in different manners to a mismatch in size involving the macrocycle cavity (2.03 ± 0.8 Å) and the metal ion [(1.19 Å) Pb(II), (0.73 Å) Cu(II), (0.95 Å) Cd(II), and (0.74 Å) Zn(II)] [71]. Actually, the bonds are able to stretch, or to compress, depending on the metal cation sizes, as it was previously reported for the metal cations under evaluation [72].

In fact, the results given in Table 2 confirm the importance of the porphyrin receptor immobilized onto silica surface on heavy metal adsorption. The free silica (**Si**) and 3-aminopropylsilica (**SiPn**) show only negligible adsorption when compared with the high metal uptake adsorption observed for **SiTF_5_PP**.

In order to understand the mechanism behind the adsorption process, a theoretical analysis of the data obtained was undertaken using equation 1 for a pseudo-first-order model and equation 2 for a pseudo-second-order model [73]:

(1) $$q_{t}{\;=\;q}_{e}\left[{1-e}^{{-k}_{1}t}\right]$$

(2) $$q_{t}=\;\frac{k_{2}q_{e}^{2}t}{{1+k}_{2}q_{e}t}$$

where q_t_ and q_e_ are the amounts of metal cations adsorbed (mg g^−1^) at time t, and at equilibrium, respectively, and k_1,_ and k_2,_ are the adsorption rate constants of the first- and second-order, respectively. The results of pseudo-first-order and pseudo-second-order parameters are given in Table 3. 

It is obvious that the regression coefficient values obtained by the pseudo-second-order model are higher for all metal cation than the ones from the pseudo-first-order model. Moreover, when it was applied the pseudo-second-order kinetics, the theoretical q_e_ values are close to the experimental values, pointing out that this model fitted well with the experimental adsorption data for Pb(II), Cu(II), Cd(II), and Zn(II). The data summarized in Table 3 also shows that the hybrid material, **SiTF_5_PP,** has a high selectivity towards Pb(II) in terms of mass quantity. In this study, we decided to adopt mass quantity and not molar quantity, since we believe that it is more traceable and facilitates the comparison with other literature studies.

#### 2.2.3. Thermodynamic Studies

**Figure 7:**
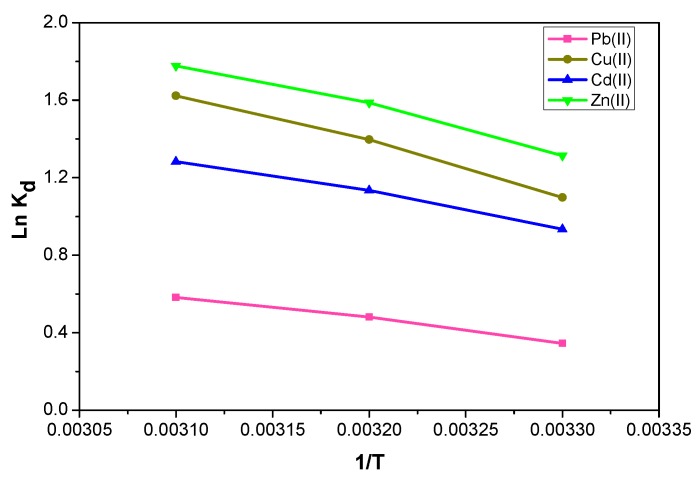
Effect of temperature for the sorption of metal ions onto **SiTF_5_PP** (contact time: 30 min; Adsorption dose: V = 10 mL, m = 10 mg of **SiTF_5_PP** using optimum pH (pH = 6), optimum concentration (187.36 ppm in each case), and 25 °C. Lines just combine the points.

The energy variation associated with the removal of Pb(II), Cu(II), Cd(II), and Zn(II) by **SiTF_5_PP,** can be determined by thermodynamic parameters [63,74,75,76,77], such as standard free energy (ΔG°; kJ mol^−1^), enthalpy (ΔH°; kJ mol^−1^), and entropy (ΔS°; kJ mol^−1^ K^−1^). These parameters were studied by carrying out the adsorption experiments between 299.15 K and 319.15 K at the optimum concentration (187.36 ppm) for each metal ion (Figure 7), and using the following equations [73]:

(3) $$K_{d}\;=\;\frac{C_{0}{-C}_{e}}{C_{e}}\frac{V}{m}\;$$

(4) $$l{\mathrm{nK}}_{d}\;=\;\frac{\Delta S{}^{\circ}}{T}-\frac{\Delta H{}^{\circ}}{\mathrm{RT}}$$

(5) ΔG° = ΔH° − TΔS° 

where C_0_ (mg L^-1^) is the initial concentration of metal solution, C_e_ (mg L^−1^) is the equilibrium concentration, V (mL) is the volume of solution, m (g) is the dosage of sorbents, R is the universal gas constant (8.314 J mol^−1^ K^−1^) and T (K) is the absolute temperature.

The ΔH°, ΔS°, and ΔG° values were calculated from the slope and intercept of ln K_d_ versus 1/T using the equations 5, 6, and 7, respectively and are summarized in Table 4. The ΔH° positive values showed that the adsorption is an endothermic process. The ΔS° positive values indicate an increase in randomness at solid-solution interface during the adsorption. The ΔG° negative values at all the studied temperatures indicate the spontaneous nature of the adsorption process for the four metal cations by the hybrid material **SiTF_5_PP** [74].

#### 2.2.4. Adsorption Isotherms

**Figure 8:**
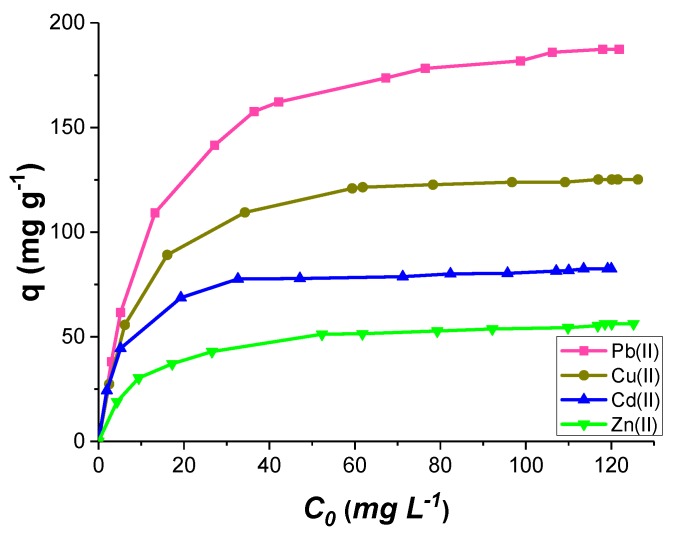
Effect of concentration on metal ions adsorption onto **SiTF_5_PP** (Adsorption dose: 10 mg; V = 10 mL; T = 25 °C; and pH = 6). Lines just combine the points.

The adsorption isotherms were determined by varying the initial concentration of metal cations from 10 to 300 mg L^−1^ with 10 mg of **SiTF_5_PP** at 25 °C and pH 6 (Figure 8). 

**Figure 9:**
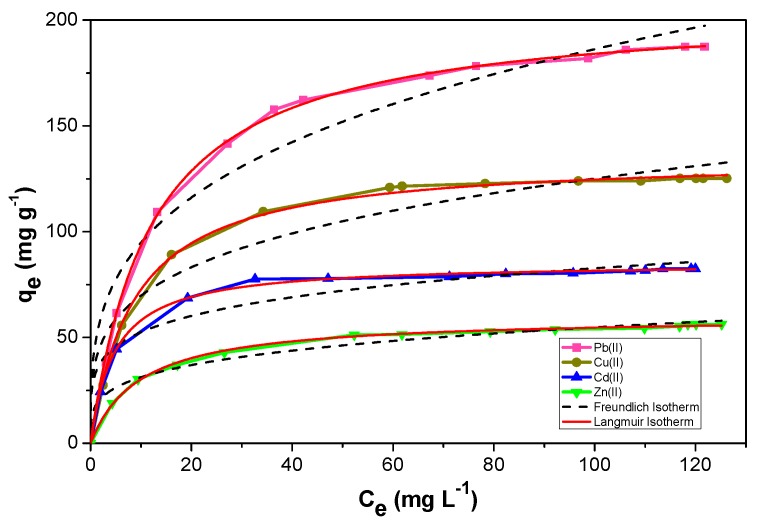
Langmuir and Freundlich adsorption models fits of Pb(II), Cu(II), Cd(II) and Zn(II) on **SiTF5PP**.

The curves of the graph show that the adsorption increases with the increase of the concentration of metal cations and reaches steady state values. The experimental data for each metal ion were fitted into both the Langmuir and the Freundlich isotherm model. The first model describes the monolayer sorption of metal ions on the surface of the sorbent, while the second one describes both multi-layer sorption and sorption on heterogeneous surfaces. The nonlinearized experimental data from the Langmuir isotherm (eqn. 6) and from the Freundlich isotherm (eqn. 7) are expressed as follows [70,78].

(6) $$q_{e}\;=\;\frac{{\mathrm{qK}}_{L}C_{e}}{{1+K}_{L}C_{e}}$$

(7) $$q_{e}{\;=\;K}_{F}C_{e}^{1/n}$$

where q_e_ is the amount of analyte sorbed (mg g^−1^), q is the saturated adsorption capacity (mg g^−1^), K_L_ is the Langmuir adsorption constant (L mg^−1^), C_e_ is the equilibrium ion concentration in the solution (mg L^−1^); K_F_ is the binding energy constant (mg g^−1^) and n is Freundlich constant. The fitted curves of nonlinear Langmuir and Freundlich models are given in Figure 9. The Langmuir and Freundlich isotherm parameters for adsorption of Pb(II), Cu(II), Cd(II) and Zn(II) are summarized in Table 5. The results show that the Langmuir isotherm fitted quite well with the experimental data (R^2^ ≥ 0.998), indicating a uniform solid surface on the sorbent, and a regular monolayer sorption. 

#### 2.2.5. Selectivity of SiTF_5_PP

**Figure 10:**
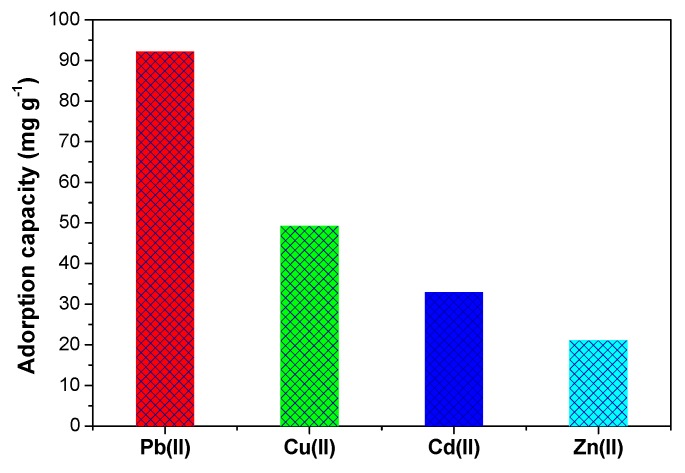
Effect of foreign metal ions on the extraction of Pb(II) with **SiTF_5_PP** (contact time: 25 min, pH = 6, T = 25 °C. Adsorption dose: V = 10 mL, m = 10 mg of **SiTF_5_PP** at optimum concentrations: 187.36 ppm of each studied metal, Pb(II), Cu(II), Cd(II), and Zn(II).

The selectivity of **SiTF_5_PP** towards Pb(II), using a mixture containing all the metal ions (Cd(II), Cu(II), Zn(II), and Pb(II); 187.36 ppm of each), was performed through a batch method, and aqueous solutions. The results are summarized in Figure 10. Although the extraction capacity seems to decrease when compared to the value obtained in the absence of extra metal cations, the outstanding selectivity observed for Pb(II) suggests that the porphyrin-silica-based hybrid **SiTF_5_PP** is a promising adsorbent, with high potential to be used to remove Pb(II) from aqueous solutions, containing competing ions.

#### 2.2.6. Applicability of SiTF_5_PP for the Removal Potentially Toxic Transition Metals from Real Samples

The applicability of **SiTF_5_PP** for the removal of Pb(II), Cu(II), Cd(II), and Zn(II) was evaluated in real conditions using natural water samples from Moulouya River (Morocco). All the samples after being collected with a polyethylene bottle, were filtered using a nylon membrane (0.45 mm) and were analyzed without storage. The assays were performed by mixing 10 mg of the adsorbent, 10 mL of the water sample, and 0.065 mL of 4.5 x 10^−3^ % HNO_3_ at 25 °C. The metal cations concentrations were determined by flame atomic absorption spectrometry (FAAS). All the experiments that were done in duplicate and are summarized in Table 6. 

For the other quality parameters of river water sample that can affect the adsorption process study, we measured the amount of several anions and cations, namely, Na^+^, Ca^2+^, K^+^, Mg^2+^, NH_4_^+^, SO_4_^2−^, NO_3_^−^, HCO_3_^−^, PO_4_^3−^ and the total organic carbon (TOC), in real water samples (see Table S2 in ESI). The results showed that under the optimum conditions established before, the presence of other ions in solution does not significantly interfere in the absorption process of the studied metal cations [Pb(II), Cu(II), Cd(II) and Zn(II)]. Therefore, the used method is suitable for analysis of real watercourse samples with complex matrixes, allowing a good affinity for Pb(II), Cu(II) and Cd(II) ions as demonstrated in this study. It is worth to refer that the presence of other ions, that naturally exist in real waters samples, present no interferences in the extraction and determination of Pb(II).

#### 2.2.7. Regeneration Ability of SiTF_5_PP

**Figure 11:**
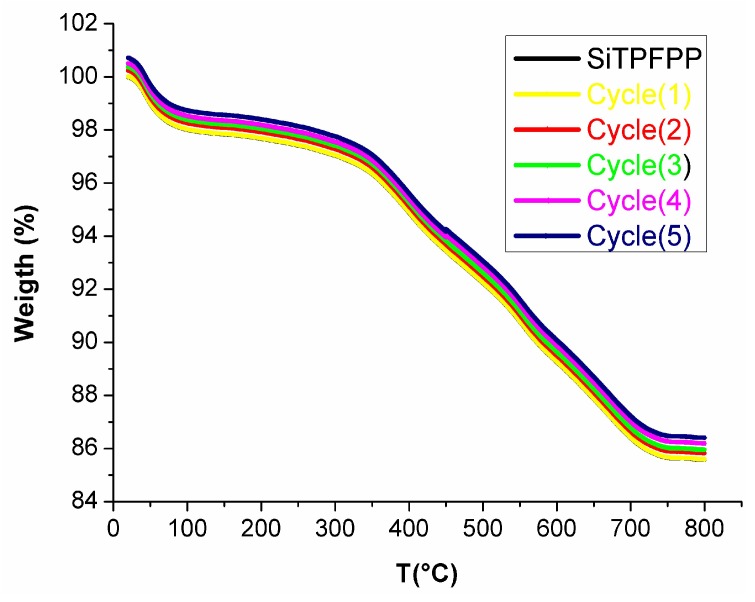
Thermogravimetric curves after five cycles of adsorbent regeneration and the curve before regeneration of **SiTF_5_PP**.

The new hybrid **SiTF_5_PP** can be easily regenerated just by washing with an acidic solution for a few minutes (6 M HCl of 5–10 mL). Indeed, after five cycles of adsorbent regeneration, no significant change in the adsorption capacity was observed (data not shown). The stability of the organic groups into the solid surface was also confirmed by TGA, with no significant alterations in the sorbent material after five utilization cycles (Figure 11).

#### 2.2.8. Comparison with Alternative Adsorbents

In Table 7 is summarized the adsorption capacity of other silica gel based adsorbents reported in the literature towards Pb(II) and the other metals. It is evident that the hybrid,, described in this work presents a higher adsorption capacity in terms of mass quantity towards Pb(II), when compared with others systems that have been recently reported in the literature and bearing a porphyrinic and non-porphyrinic ligands. The results in Table 7 also show that the new silica-based adsorbent functionalized with a porphyrin unit prepared in this work, has a remarkable adsorption ability for Cu(II) ion. 

## 3. Materials and Methods 

### 3.1. General Remarks 

All solvents and other chemicals (Aldrich, Steinheim, Germany, purity > 99.5%) were of analytical grade and used without further purification. Silica gel (Merck, Darmstadt, Germany; 70–230 mesh, 60 Å), was activated before use at 120 °C for 24 h. The 3-aminopropyltrimethoxysilane (Janssen Chemicals, Beerse, Belgium) was used without purification. The silica particles were characterized on a scanning transmission electron microscope (SEM) Hitachi S4100 (Hitachi, Krefeld, Germany), equipped with an energy dispersive spectrometer operating at 30 kV and a Jeol JEM-2200FS (JEOL, Tokyo, Japan) transmission electron microscope (TEM) operating at 200 kV. A Micromeritics Gemini 2380 surface area analyzer with *ca.* 50 mg weight was used to perform the Brunauer-Emmett-Teller (BET)/Barrett-Joyner-Halenda (BJH) determination of specific surface area and pore size distribution. The ^13^C solid-state CPMAS NMR spectroscopy experiments were recorded on a Bruker Avance III 400 spectrometer (Wissembourg, France) at room temperature, using a double-resonance 4 mm Bruker MAS probe and a spinning rate of 12 kHz. The ^13^C Larmor frequency was 100.6 MHz. Elemental analyses (EA) were performed on a LECO CHNS-932 apparatus (LECO Corporation, MI, USA). Attenuated Total Reflectance Transmission Fourier Transform Infrared (ATR-FTIR) spectra were recorded on a FT Mattson 7000 galaxy series spectrophotometer (Mattson Instruments, WI, USA). Solid UV-Vis absorption spectra in the spectral range 350–800 nm were registered using a JASCO V-560 spectrophotometer (JASCO International Co., Ltd., Tokyo, Japan). For the X-ray diffraction (XRD) measurements, self-oriented solids were placed on neutral glass sample holders. The measurements were done in the reflection mode using X’Pert MPD Philips diffractometer (Cu Kα1,2 X-radiation, λ_1_ = 1.540598 Å and λ_2_ = 1.544426 Å, (Malvern, Gondomar, Portugal), equipped with an X’Celerator detector and a flat plate sample holder in a Bragg-Brentano para-focusing optics configuration (40 kV, 50 mA). Intensity data were collected by the step counting method (step 0.02), in continuous mode. The nitrogen adsorption-desorption was obtained using a Thermoquest Sorpsomatic 1990 analyzer (Micromeritics Headquarters, GA, USA). The mass loss assays were performed in 90:10 oxygen/nitrogen atmospheres on a TGA Q50 V6.7 Build 203 instrument (Shimatzu, Kyoto, Japan), at a heating rate of 10 °C min^−1^. The metal ions determination was performed by atomic absorption measurements, using a Spectra Varian A.A. 400 spectrophotometer (COSTE, Oujda, Morocco), equipped with an air-acetylene flame. The wavelength used for monitoring the transitions metals Pb(II), Cu(II), Cd(II,) and Zn(II) is 283.3, 324.8, 228.8 and 213.9 nm, respectively. Metal cations detection is in the range: 1–12 ppm, for Pb, 1–4 ppm for Cu, and 0.1–0.6 ppm for Cd, and Zn, respectively. The calibration curve method was used to elucidate the results of measurements.

### 3.2. Preparation of 3-Aminopropylsilica (SiPn) 

A dispersion of activated silica gel (30 g) in dry toluene (200 mL) was refluxed and mechanically stirred under a nitrogen atmosphere for 2 h. Then, under continuous stirring, it was added 3-aminopropyltrimethoxysilane (13 mL) gradually and the mixture was refluxed for 24 h. The resulting solid was filtered washed with toluene and ethanol and Soxhlet extracted with a mixture of ethanol and dichloromethane (1/1) for 12 h, to remove the silylating reagent residue [81,94]. The functionalized silica gel, named **SiPn**, was dried at room temperature for 1 h under vacuum and characterized by elemental analysis. Elemental analyses: %C = 5.03; %N = 1.63.

### 3.3. Synthesis of Porphyrin-Substituted Silica (SiTF_5_PP)

A mixture containing **SiPn** (4 g) and 150 mg of 5,10,15,20-tetrakis(pentafluorophenyl)porphyrin **H_2_TF_5_PP** in 30 mL of dry toluene was maintained under reflux for 48 h. Then, the resulting solid was filtered, dried and Soxhlet extracted with toluene, methanol and dichloromethane for 12 h. The product named **SiTF_5_PP** was then dried at 40 °C for 4 h under vacuum. Elemental analyses: %C = 8.00; %N = 1.53.

### 3.4. Metal Cation Adsorption Experiments

The experiments to evaluate the metal adsorption ability of the synthesized hybrid material towards Pb(II), Cu(II), Cd(II), and Zn(II) were performed at 25 °C by stirring 10 mg of **SiTF_5_PP,** with 10 mL of an aqueous solution containing a single-metal at the optimum concentration of 187.36 ppm. The pH of the solution was adjusted using 0.1 M HCl and 0.1 M NaOH solutions. After shaking for 1 h, the adsorbent-solution mixtures were filtered to collect the final solutions. The initial and final metal concentrations (before and after the presence of the adsorbent material **SiTF_5_PP)** were determined by the flame atomic absorption spectrometry (FAAS). The amount of metal ions adsorbed by **SiTF_5_PP** from aqueous solution was calculated using the following equations [95]:

(8) $$Q_{M}\;=\;\left(C_{0}{-C}_{e}\right)\times V/W\;$$

(9) $$Q_{W\;}{=\;Q}_{M}\times M\;$$

where Q_M_ is the amount of the metal ion on the adsorbent (mmol g^−1^), Q_W_ is the amount of the metal ion on the adsorbent (mg g^−1^), V is the volume of the aqueous solution (L), W is the weight of the adsorbent (g), C_0_ is the initial concentration of metal ion (mmol L^−1^), C_e_ is the equilibrium metal ion concentration in solution (mmol L^−1^), and M the atomic weight of the metal (g mol^−1^).

### 3.5. Batch Experiments

The applicability of **SiTF_5_PP** for the removal of transition metals [Pb(II), Cu(II), Cd(II), and Zn(II)]) was determined by adding 10 mg of the adsorbent to an aqueous solution of each metal ion (10 mL) at different concentrations (10 to 300 mg L^−1^) at 25 °C. The pH effect was studied in the range of 1−7. The effect of contact time and kinetic modelling were also determined at room temperature for 5−30 min. After a pre-established contact time, the mixture was then filtered and the unextracted metal ion present in the filtrate (supernatant) was determined using a flame atomic absorption spectrometer (FAAS). Analyses were performed in duplicate for each sample, and the mean data were reported.

## 4. Conclusions

In summary, this work allowed the development and characterization of an efficient inorganic-organic hybrid material for metal sorption, based on the heterogenization of 5,10,15,20-tetrakis(pentafluorophenyl)porphyrin **H_2_TF_5_PP** on a chemically modified silica.

The study shows that metal cations adsorption is more efficient at pH in the range 5 to 7, and the maximum adsorption is reached in only 25 min, suggesting a fast-external diffusion and surface adsorption. The hybrid **SiTF_5_PP** is particularly efficient towards Pb(II) with a maximum absorption value of 187.36 mg g^−1^, while for the others metal cations studied were obtained values in the range 125.17–56.23 mg g^−1^. Its efficiency can be attributed to the presence of fluorine atoms on the *meso* phenyl rings of the porphyrinic macrocycle, which increase the electron density of the nitrogen in the core.

The adsorption kinetics fit into the pseudo-second-order model, showing homogeneous characteristics. The comparison of different isotherm models indicated that the Langmuir model gave the best fit to the experimental data, and the increase of the metal cations adsorption ability with the temperature, indicates that this is an endothermic and spontaneous process.

The hybrid prepared displays an excellent adsorption ability towards Pb(II) and Cu(II), when compared with other adsorbents described in the literature, and showed a high performance in a competitive mode and in real water samples. Moreover, it can be regenerated several times (at least 5 times) without the loss of their adsorption capability, suggesting that an efficient and low-cost adsorbent for metal cations removal from aqueous solutions.

## Figures and Tables

**Table 1:** Physical properties of silica-based materials.

Materials	Specific Surface S*_BET_* (m^2^ g^−1^)	Pore Volume (cm^3^ g^−1^)	Average Pore Diameter (Å)
**Si**	434.6526 ± 4.6197	0.671 ± 0.002	47.882
**SiPn**	328.7584 ± 5.5689	0.482 ± 0.003	46.358
**SiTF_5_PP**	341.8908 ± 4.0805	0.477 ± 0.010	46.307

**Table 2:** Metal ion adsorption of **Si**, **SiPn** and **SiTF_5_PP** as contact time (30 min) function.

Material	*q_e_* ± 1.0 (mg g^−1^)			
	Pb(II)	Cu(II)	Cd(II)	Zn(II)
**Si**	4.35	4.58	3.26	6.89
**SiPn**	6.34	5.32	6.57	7.86
**SiTF_5_PP**	187.36	125.16	82.44	56.23

**Table 3:** Kinetics of heavy metals removal onto **SiTF_5_PP**.

Parameters	Metals			
	Pb(II) (1.19 Å)	Cu(II) (0.73 Å)	Cd(II) (0.95 Å)	Zn(II) (0.74 Å)
q_e_ (exp) (mg g^−1^)	187.36 ± 1	125.17 ± 1	82.45 ± 1	56.23 ± 1
q_e_ (exp) (mmol g^−1^)	0.90	1.97	0.73	0.86
	Pseudo-first-order			
q_e_ (mg g^−1^)	182.695 ± 2.095	123.393 ± 1.223	81.634 ± 0.568	55.390 ± 0.412
q_e_ (mmol g^−1^)	0.881	1.943	0.726	0.847
k_1_ (min^−1^)	0.447 ± 0.50	0.340 ± 0.024	0.365 ± 0.019	0.322 ± 0.016
R^2^	0.996	0.997	0.998	0.998
	Pseudo-second-order			
q_e_ (mg ^−1^g)	191.205 ± 1.613	132.122 ± 0.651	83.641 ± 0.572	59.644 ± 0.455
q_e_ (mmol g^−1^)	0.922	2.080	0.744	0.912
k_2_ (g mg^−1^ min^−1^)	(6.11 ± 0.77) 10^−^^3^	(5.27 ± 0.26) 10^−^^3^	(9.40 ± 0.70) 10^−^^3^	(10.53 ± 0.75) 10^−^^3^
R^2^	0.999	0.999	0.999	0.999

**Table 4:** Adsorption isotherm parameters of metal ions onto **SiTF_5_PP**.

Metal	ΔH° (kJ mol^−1^)	ΔS° (J K^−1^ mol^−1^)	T (K) ± 1 K	ΔG° (kJ mol^−1^)
Pb(II)	9.883 ± 0.100	35.52 ± 0.321	299.15 309.15 319.15	−0.742 −1.098 −1.453
Cu(II)	21.834 ± 0.206	81.26 ± 0.659	299.15 309.15 319.15	−2.477 −3.290 −4.103
Cd(II)	14.485 ± 0.149	55.63 ± 0.477	299.15 309.15 319.15	−2.158 −2.714 −3.271
Zn(II)	19.275 ± 0.237	74.50 ± 0.759	299.15 309.15 319.15	−3.010 −3.756 −4.500

**Table 5:** Adsorption isotherm parameters of metal cations onto **SiTF_5_PP**.

Metal	Langmuir Isotherm Model				Freundlich Isotherm Model		
	q (mg g^−1^)	q (mmol g^−1^)	K_L_ (L mg^−1^)	R^2^	K_F_ (mg g^−1^)	N	R^2^
Pb(II)	206.282 ± 0.992	0.995	0.082 ± 0.001	0.999	48.209 ± 6.486	3.407 ± 0.383	0.952
Cu(II)	135.374 ± 0.880	2.131	0.115 ± 0.004	0.998	38.920 ± 5.789	3.945 ± 0.529	0.944
Cd(II)	85.384 ± 0.444	0.759	0.212 ± 0.008	0.998	33.282 ± 4.043	5.060 ± 0.723	0.944
Zn(II)	59.997 ± 0.400	0.917	0.101 ± 0.003	0.998	17.724 ± 1.637	4.080 ± 0.354	0.974

**Table 6:** Extraction of potential toxic metal cations in natural real water samples.

Water Samples	Metal Ion	C_found_ ± 0.05 (mg L^−1^)	Metal Ions Concentration after Adsorption on SiTF_5_PP (mg g^−1^)
Moulouya River	Pb(II)	0.7506	0.6199
	Cd(II)	0.3133	0.2048
	Cu(II)	0.9794	0.1726
	Zn(II)	Not detectable	-

**C_found_** is the initial metal contents in river water determined by atomic absorption measurements.

**Table 7:** Comparison of **SiTF_5_PP** performances with some recently reported sorbents for Pb(II) adsorption.

Silica gel - Ligand	Ref	Adsorption Capacity (mg g^−1^)			
		Pb(II)	Cu(II)	Cd(II)	Zn(II)
5,10,15,20-Tetrakis(pentafluorophenyl)porphyrin	This work	187.36	125.17	82.45	56.23
5,10,15,20-Tetraphenylporphyrin	[68]	55.17	19.08	26.46	34.62
*N,N*-bipyrazole amine	[25]	123	-	-	-
Glutaraldehyde	[79]	29.32	-	-	-
2-Hydroxy-3-methoxybenzaldehyde	[80]	2.27	4.70	-	-
Bipyridine tripodal receptor	[81]	99.68	64.84	42.18	82.68
Nitrothiophene	[82]	52.41	61.52	38.45	35.72
PMAEEDA	[83]	61.90	19.96	19.34	17.16
Ketoenol Furane	[84]	18.75	32.08	52.15	23.36
*N*-[3(Trimethoxysilyl)propyl]ethyl-ene-diamine	[85]	96.43	27.22	-	12.16
3-(2-Aminoethylamino)pro-pyldimethoxymethylsilane	[86]	100.58	74.69	121.43	-
SiO_2_-g-GMA/β-CD-Ac.	[87]	51.02	110.0	15.20	-
Pentane-1,2-dicarboxylic acid	[88]	122.0	38.0	30.0	28.0
(*E*)-4-((Pyridin-2-yl-methyl-ene)amino)phenol	[29]	36.38	-	32.38	92.00
Dithiocarbamate	[89]	42.16	25.00	10.01	26.01
Cetyltrimethylammonium bromide	[90]	-	32.20	8.00	-
Nano-sized Fe_3_O_4_	[91]	166.67	37.04	-	-
MCM-41/*N*-(3-trimethoxysilyl)-propyl) diethylenetriamine	[92]	77.52	-	-	-
(*2Z*)-1-(1,5-dimethyl-1H-pyrazol-3-yl)-3-hydroxybut-2-en-1-one	[93]	94.18	71.40	40.18	83.33

## Supplementary Materials

The following are available online, Table S1: Elemental analysis, Figure S1: ATR-FTIR Spectra of free silica (**Si**), 3-aminopropylsilica (**SiPn**) and **SiTF_5_PP**, Figure S2: ^13^C NMR spectra of **SiPn** (top) **SiTF_5_PP** (bottom), Figure S3: X - ray diffraction spectra of free silica (**Si**), 3-aminopropyl-silica (**SiPn**) and **SiTF_5_PP**, Figure S4: Electronic density for **NTPP** and **H_2_(TF_5_PP)** determinate with Marvin 6.1.6. Software.
