# Supplementary material for: Supramolecular Hybrid Material Based on Engineering Porphyrin Hosts for an Efficient Elimination of Lead(II) from Aquatic Medium
Source: Molecules. 2019 Feb 14;24(4):669. doi: 10.3390/molecules24040669 (PMC6412391; doi:10.3390/molecules24040669)
Supplement: Supplementary file 1 [file molecules-24-00669-s001.pdf]

Electronic Supporting Information

Supramolecular Hybrid Material Based on Engineering Porphyrin Hosts for an Efficient Elimination of Lead(II) from Aquatic Medium

Chahrazad El Abiad [1], Smaail Radi [1,*], Maria A. F. Faustino [2], M. Graça P. M. S. Neves [2], Nuno M. M. Moura [2,*]

[1] Laboratory of Applied Chemistry and Environment (LCAE), Department of Chemistry, Faculty of Sciences, University Mohamed Premier, Oujda, Morocco

[2] QOPNA & LAQV-REQUIMTE, Department of Chemistry, University of Aveiro, 3810-193 Aveiro, Portugal

Table S1. Elemental analysis.

Sample	C%	N%
**SiPn**	5.03	1.63
**SiTF$_5$PP**	8.00	1.53

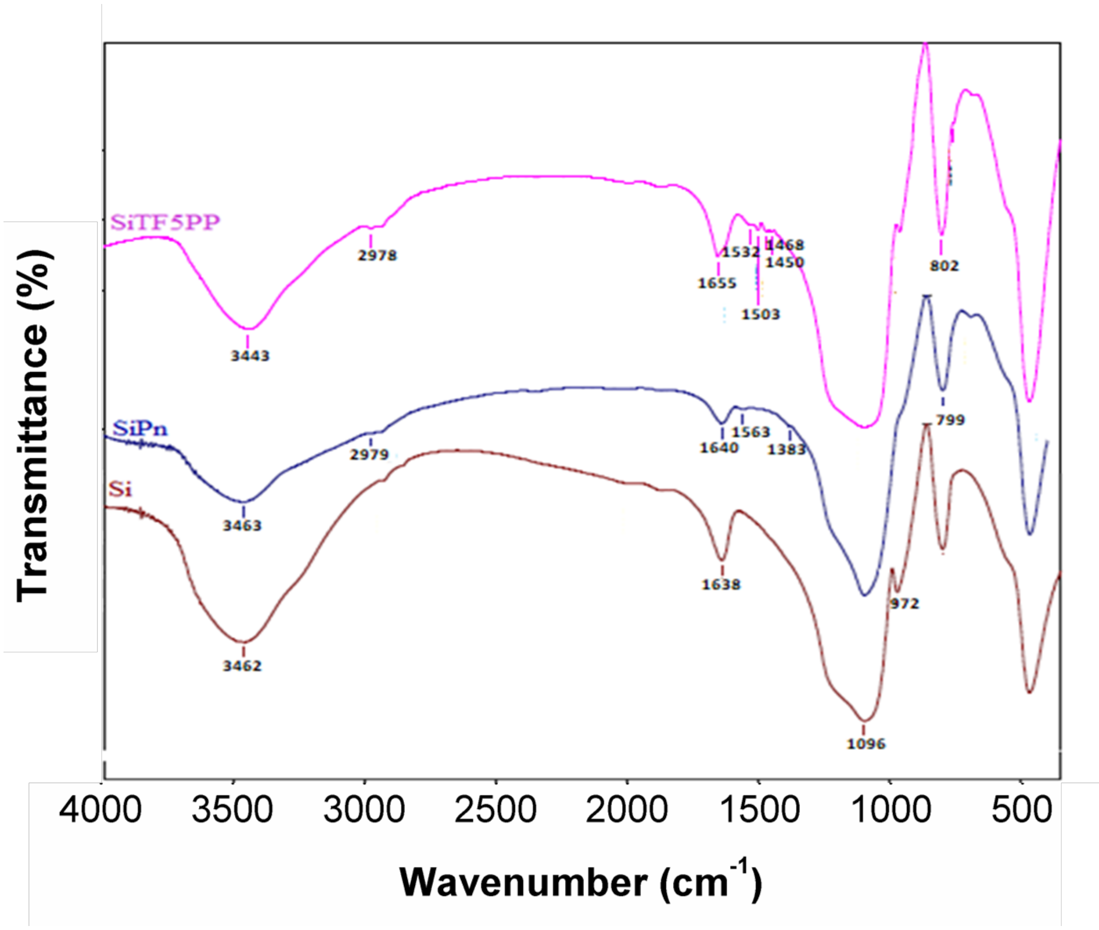

Figure S1. ATR-FTIR Spectra of free silica (**Si**), 3-aminopropylsilica (**SiPn**) and **SiTF$_5$PP**.

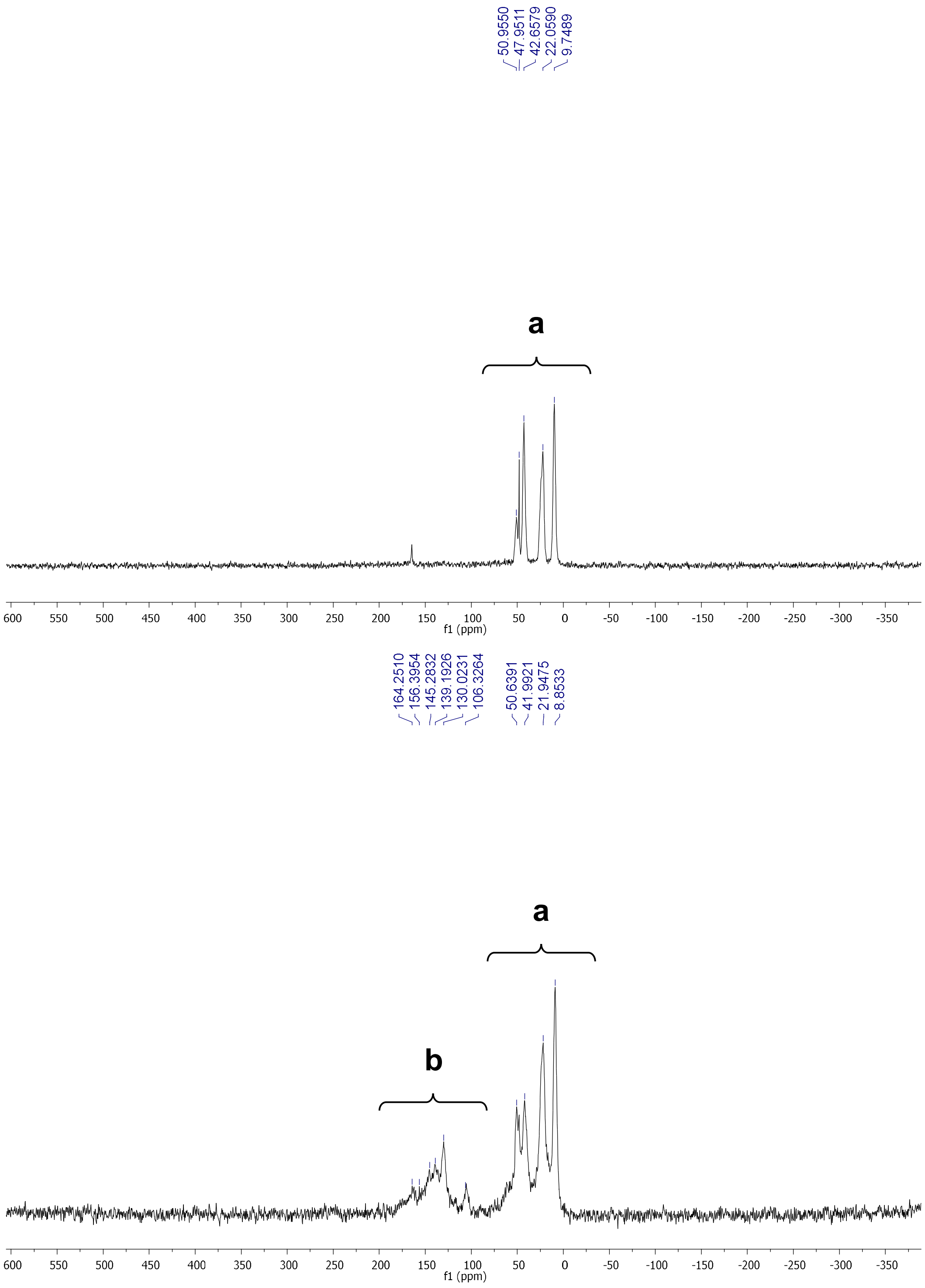

Figure S2. ^{13}C NMR spectra of **SiPn** (top) **SiTF$_5$PP** (bottom).

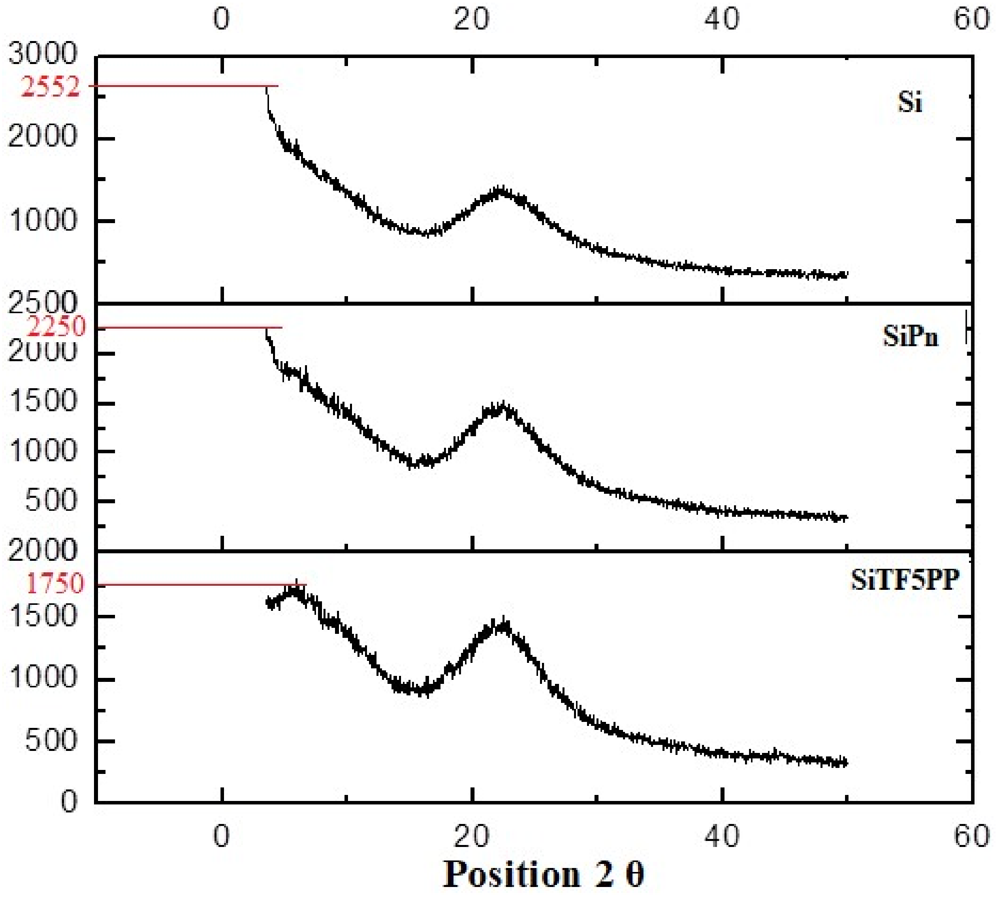

Figure S3. X - ray diffraction spectra of free silica (**Si**), 3-aminopropyl-silica (**SiPn**) and **SiTF$_5$PP**.

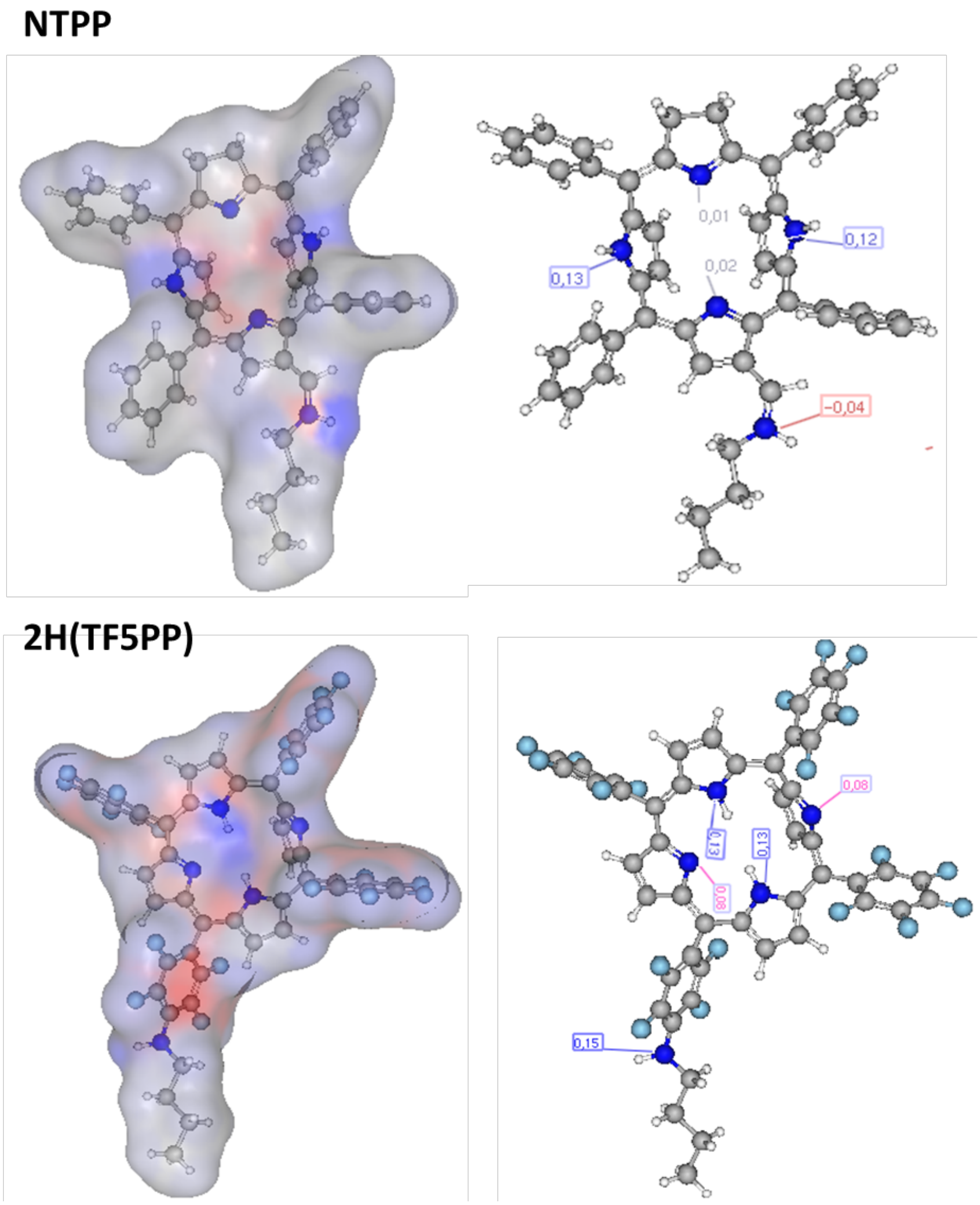

Figure S4. Electronic density for **NTPP** and **2H(TF5PP)** determinate with Marvin 6.1.6. Software.

Table S2. Quality parameters of Moulouya River water.

Sample	Ion	Concentration (mg L^{-1})
Moulouya River	Na^+	341.30
	K^+	15.00
	Mg^{2+}	70.67
	Ca^{2+}	304.60
	NH_4^+	0.167
	SO_4^{2-}	528.25
	HCO_3^-	280.60
	NO_3^-	25.69
	PO_4^{3-}	0.29
	TOC	8.59
